# Midwives and the National Insurance Bill

**Published:** 1911-06-03

**Authors:** 


### MIDWIVES AND THE NATIONAL INSURANCE BILL.

The Incorporated Midwives' Institute, of 12 Buckingham Street, Strand, W.C., in a letter to the Press, points out that there are 30,000 women on the Midwives Roll, and the Midwives' Institute, being the only incorporated body of midwives, feels bound to speak on behalf of this large number of useful members of the community, who are unable themselves to voice their views. Some 50 per cent. of the total number of births in England and Wales are attended by midwives: the percentage will necessarily be much higher amongst the class insured under Mr. Lloyd George's Bill. In one part of the Bill it is stated that the woman entitled to maternity benefit shall not be entitled to sickness or medical benefit for four weeks after her confinement. In answer to a question put by Mr. Lees Smith, and reported by the *Daily Telegraph*, Mr. Lloyd George said that the maternity benefit under the Bill (which covered medical attendance) must be regarded as additional to sickness benefit and not as a substitute for it. If this means that a woman can be entitled to sickness benefit in addition to maternity benefit, it will be a great relief to many poor women to know it, and it would leave the 30s. (which, by the by, is not apportioned by the woman who has paid for it, but by a Health Committee, as may be prescribed), presumably for medical attendance and extras, including nursing. The Institute suggests that it should be laid down in the Act that the lying-in woman shall have entirely free choice as to whether she employs a doctor or a midwife, and liberty to choose that doctor or midwife; also, if she employs a midwife, and it is necessary for the midwife to send for a doctor, that his fee shall be assured. The medical profession is able through its powerful organisations to influence Parliament. The midwife is, by reason of her sex, excluded from any participation in making the laws that concern her, and the only hope of obtaining a small modicum of justice is by appealing to the public.

